# Repurposing chemotherapy‐induced peripheral neuropathy grading

**DOI:** 10.1111/ene.16457

**Published:** 2024-09-16

**Authors:** Roser Velasco, Andreas A. Argyriou, David R. Cornblath, Pere Bruna, Paola Alberti, Emanuela Rossi, Ingemar S. J. Merkies, Dimitri Psimaras, Chiara Briani, Roy I. Lalisang, Angelo Schenone, Guido Cavaletti, Jordi Bruna, CI‐PeriNomS Group, G. Cavaletti, B. Frigeni, F. Lanzani, L. Mattavelli, M. L. Piatti, P. Alberti, D. Binda, P. Bidoli, M. Cazzaniga, D. Cortinovis, J. Bruna, R. Velasco, A. A. Argyriou, H. P. Kalofonos, D. Psimaras, D. Ricard, A. Pace, E. Galiè, C. Briani, M. Lucchetta, M. Campagnolo, C. Dalla Torre, C. G. Faber, I. S. J. Merkies, E. K. Vanhoutte, M. Bakkers, B. Brouwer, M. Boogerd, R. I. Lalisang, W. Boogerd, D. Brandsma, S. Koeppen, J. Hense, R. Grant, D. Storey, S. Kerrigan, A. Schenone, M. Belllucci, A. Pessino, L. Padua, G. Granata, M. Leandri, I. Ghignotti, R. Plasmati, F. Pastorelli, T. J. Postma, J. J. Heimans, M. Eurelings, R. J. Meijer, W. Grisold, E. LindeckPozza, A. Mazzeo, A. Toscano, M. Russo, C. Tomasello, G. Altavilla, M. Penas‐Prado, C Dominguez‐Gonzalez, S. G. Dorsey

**Affiliations:** ^1^ Unit of Neuro‐Oncology Hospital Universitari de Bellvitge‐Institut Català Oncologia, Bellvitge Institute for Biomedical Research, L'Hospitalet de Llobregat Barcelona Spain; ^2^ Neurological Department Agios Andreas General Hospital of Patras Patras Greece; ^3^ Department of Neurology, School of Medicine Johns Hopkins University Baltimore Maryland USA; ^4^ Department of Physics, Barcelona Research Center in Multiscale Science and Engineering Universitat Politècnica de Catalunya (UPC), BarcelonaTech, Institut de Tècniques Energètiques Barcelona Spain; ^5^ Department of Neuroscience and Biomedical Technologies University of Milano‐Bicocca Monza Italy; ^6^ Center of Biostatistics for Clinical Epidemiology, Department of Clinical Medicine and Prevention University of Milano‐Bicocca Monza Italy; ^7^ Department of Neurology Maastricht University Medical Center+ Maastricht Limburg the Netherlands; ^8^ Institute of Neurology, Unité INSERM U1127, CNRS UMR 7225 (Institut du Cerveau et de la Moelle épinière) and OncoNeuroTox Group, Center for Patients With Neurological Complications of Oncologic Treatments Hôpitaux Universitaires la Pitié Salpêtrière Paris France; ^9^ Department of Neurosciences University of Padua Padua Italy; ^10^ Division of Medical Oncology, Department of Internal Medicine, GROW‐School of Oncology and Developmental Biology Maastricht University Medical Center Maastricht the Netherlands; ^11^ Department of Neurosciences, Rehabilitation, Ophthalmology, Genetic and Maternal and Infantile Sciences University of Genoa and IRCCS San Martino Hospital Genoa Italy

**Keywords:** chemotherapy, chemotherapy‐induced peripheral neuropathy, neurotoxicity, patient‐reported outcome measure

## Abstract

**Background and Purpose:**

Chemotherapy‐induced peripheral neuropathy (CIPN) is perceived differently by patients and physicians, complicating its assessment. Current recommendations advocate combining clinical and patient‐reported outcomes measures, but this approach can be challenging in patient care. This multicenter European study aims to bridge the gap between patients' perceptions and neurological impairments by aligning both perspectives to improve treatment decision‐making.

**Methods:**

Data were pooled from two prospective studies of subjects (*n* = 372) with established CIPN. Patient and physician views regarding CIPN were assessed using the National Cancer Institute Common Terminology Criteria for Adverse Events (NCI‐CTCAE), Total Neuropathy Scale–clinical version (TNSc) items, and the disease‐specific quality of life ‐ Chemotherapy‐Induced Peripheral Neuropathy questionnaire (QLQ‐CIPN20) from the European Organization for Research and Treatment of Cancer (EORTC). To identify inherent neurotoxic severity patterns, we employed hierarchical cluster analysis optimized with k‐means clustering and internally validated by discriminant functional analysis.

**Results:**

Both NCI‐CTCAE and TNSc demonstrated a significant difference in the distribution of severity grades in relation to QLQ‐CIPN20 scores. However, a proportion of subjects with different neurotoxic severity grades exhibited overlapping QLQ‐CIPN20 scores. We identified three distinct clusters classifying subjects as having severely impaired, intermediately impaired, and mildly impaired CIPN based on TNSc and QLQ‐CIPN20 scores. No differences in demographics, cancer type distribution, or class of drug received were observed.

**Conclusions:**

Our results confirm the heterogeneity in CIPN perception between patients and physicians and identify three well‐differentiated subgroups of patients delineated by degree of CIPN impairment based on scores derived from TNSc and QLQ‐CIPN20. A more refined assessment of CIPN could potentially be achieved using the calculator tool derived from the cluster equations in this study. This tool, which facilitates individual patient classification, requires prospective validation.

## INTRODUCTION

There are significant challenges to correctly assessing and interpreting chemotherapy‐induced peripheral neurotoxicity (CIPN), primarily due to the differing perceptions of this clinically relevant toxicity by patients and physicians [1]. Accurate grading of CIPN is essential for making informed decisions regarding the management of drug regimens during cancer treatment and assessing their long‐term consequences. Although the National Cancer Institute Common Terminology Criteria for Adverse Events (NCI‐CTCAE) is the most widely used scale in oncology for evaluating adverse events, including neurotoxicity, there are limitations in its assessment of CIPN. Notably, NCI‐CTCAE's peripheral neuropathy scale emphasizes the impact of neurological symptoms on patients' functionality. However, the relationship between this clinical reported outcome (CRO) scale and patients' perception is not consistent, particularly in CIPN of intermediate severity [1]. Furthermore, the interpretation of the origin of CIPN symptoms, even by experienced oncologists, often does not align with objective neurological impairment [2].

In contrast, neurologists have introduced the Total Neuropathy Scale–clinical version (TNSc), an alternative CRO scale specifically designed to address CIPN severity [3]. Compared with NCI‐CTCAE, TNSc provides measurable detailed objective clinical neurological data and exhibits slightly better clinimetric properties [4]. In contrast with NCI‐CTCAE, TNSc, which also operates as an ordinal scale, places greater emphasis on objective neurological examination data, potentially diminishing the symptomatic aspects. Furthermore, the categorization of TNSc into discrete groups corresponding to different severities of neuropathy has not been sufficiently explored and presents challenges in practical clinical decision‐making.

The variations among CROs and the different perspectives on neurotoxicity offered by patient‐reported outcomes (PROs) underscore the need for a standardized set of integrative outcome measures for CIPN management. To address this clinical unmet need, we conducted a multicenter European study with the specific goal of bridging the gap between patients' perceptions of CIPN and the severity of neurological impairments quantified by TNSc. We aimed to identify distinct patient clusters based on TNSc variables and quality of life test scores. This approach aims to facilitate the development of a pragmatic severity grade categorization that can effectively guide clinical decisions during cancer treatment, thereby enabling a potentially more accurate classification of the enduring consequences of CIPN.

## METHODS

### Patient sample

Data were collected and pooled from two prospective studies of patients with established CIPN: 281 subjects who participated in the initial assessment of the multicenter European CI‐PeriNomS study [4] and 102 subjects who participated in a study conducted at Hospital Universitari de Bellvitge–ICO L'Hospitalet assessing sarcopenia as a risk factor for developing CIPN. Our study included those participants who had established CIPN and had undergone assessments related to quality of life and neurological symptoms, as well as NCI‐CTCAE evaluations. Overall, 372 subjects were included in our study: 281 from the CI‐PeriNomS study and 91 from the Hospital Universitari de Bellvitge–ICO L'Hospitalet study.

During the conduct of the two original studies, ethical approvals were obtained from institutional review boards (IRBs) at participating centers and written informed consent was obtained from all participants. This secondary analysis was aligned with the objectives of CI‐PeriNomS and covered under the IRB approvals for the study. A new approval was obtained from the Ethics Committee of Hospital Universitari de Bellvitge–ICO L'Hospitalet to analyze the data for this study (PR321/20).

Anonymized data not published within this article will be made available by request from any qualified investigators. The STROBE (Strengthening the Reporting of Observational Studies in Epidemiology) checklist for cohort and cross‐sectional studies is provided in Appendix S1.

### Inclusion and exclusion criteria

Participants were included if they were 18 years or older, had a Karnofsky Performance Status of ≥70, and had received a noninvestigational neurotoxic drug and subsequently developed CIPN. CIPN was defined as the presence of typical symptoms and signs of chemotherapy dose‐related polyneuropathy that were absent prior to chemotherapy treatment. Exclusion criteria included any factors that could potentially confound the assessment of CIPN, such as peripheral damage related to any other cause, concurrent use of neurotoxic medications, other coexisting medical conditions, and neurological disorders that could complicate the accurate interpretation of results.

### Study design and assessment methods

Trained investigators at each participating center administered the NCI‐CTCAE scale for neurotoxicity, TNSc, and the disease‐specific Quality of Life ‐ Chemotherapy ‐Induced Peripheral Neuropathy questionnaire (QLQ‐CIPN20) from the European Organization for Research and Treatment of Cancer (EORTC) [5]. Data collected after completion of chemotherapy treatment were used for this study. For participants from the CI‐PeriNomS study, there was a prerequisite that they maintain a stable neurological condition for at least 2 months after finishing their chemotherapy schedule prior to study participation. For participants from the Hospital Universitari de Bellvitge–ICO L'Hospitalet study, data corresponded to their final assessment conducted 1–3 months following chemotherapy treatment.

The QLQ‐CIPN20 consists of 20 items, rated by subjects on a 4‐point Likert‐type scale ranging from 1 ("not at all") to 4 ("very much"). The final score was calculated in accordance with standard EORTC scoring procedures (https://www.eortc.org/app/uploads/sites/2/2018/02/SCmanual.pdf). Recent psychometric evaluations prompted the exclusion of items 19 and 20, with a straightforward additive scoring procedure implemented instead [6, 7].

TNSc is a well‐validated version of the TNS [8] specifically designed for assessing patients with CIPN without involving nerve conduction or quantitative vibration threshold evaluation [3]. The scale comprises seven items assessing symptoms (sensory [S], motor [M], and autonomic [A]) and signs (reflexes [R], vibration [V], pin sensitivity [P], and muscle strength [St]). Each item is rated from 0 to 4, contributing to a single measure obtained by summing the scores from each item to eventually quantify the severity of CIPN. To ensure balanced weighting, TNSc was divided into two subscores, one for symptoms (SMA) and one for signs (RVPSt).

### Cluster and statistical analysis

A two‐stage approach to cluster analysis was employed to identify inherent neurotoxic severity patterns from the datasets, using TNSc items and QLQ‐CIPN20 scores. The hierarchical analysis used the single linkage method to avoid imposing any preconceived cluster structures. Specifically, an unsupervised agglomerative hierarchical analysis first explored the potential number of groups followed by a nonhierarchical k‐means analysis to perform the full clustering. The squared Euclidean distance was used as the distance measure for cluster observations. The number of clusters was determined through inspection of the dendrogram and the agglomeration schedule coefficients in the scree plot. Subsequently, a k‐means analysis was conducted to optimize the retained number of clusters. This algorithm partitioned the data into k distinct clusters based on the proximity of data points to the centroids. The centroid represented the average of variables computed exclusively for observations within the cluster, and the distance between the observations and the centroid was reduced to the Euclidean distance. The algorithm iteratively adjusted the assignment of data points to minimize the distance of individual observations from the centroid of a cluster while simultaneously maximizing the distance from the centroids of other clusters. This iteration continued until convergence was reached, ensuring stability in the cluster assignments. Notably, the number of clusters determined from the hierarchical analysis served as our input parameter in this phase. Subsequently, discriminant function analysis (DFA) was carried out to assess the internal validity of the clustering solution and determine the predictive power of the TNSc items and QLQ‐CIPN20 scores in differentiating patients into neurotoxic severity subgroups [9]. Leave‐one‐out classification evaluated the reliability of the DFA‐generated model. Demographic and clinical characteristics of cluster subjects were compared using Kruskal–Wallis and analysis of variance followed by post hoc Bonferroni tests. Descriptive data analysis presented categorical variables as observed count and weighted percentage, whereas continuous variables were expressed as mean or median along with corresponding SE or range. All analyses were conducted using SPSS software package V.23.0 (SPSS, Chicago, IL, USA), with *p*‐values < 0.05 considered statistically significant.

## RESULTS

### Relationship between CRO neurotoxicity scales and PRO QLQ‐CIPN20

**FIGURE 1:**
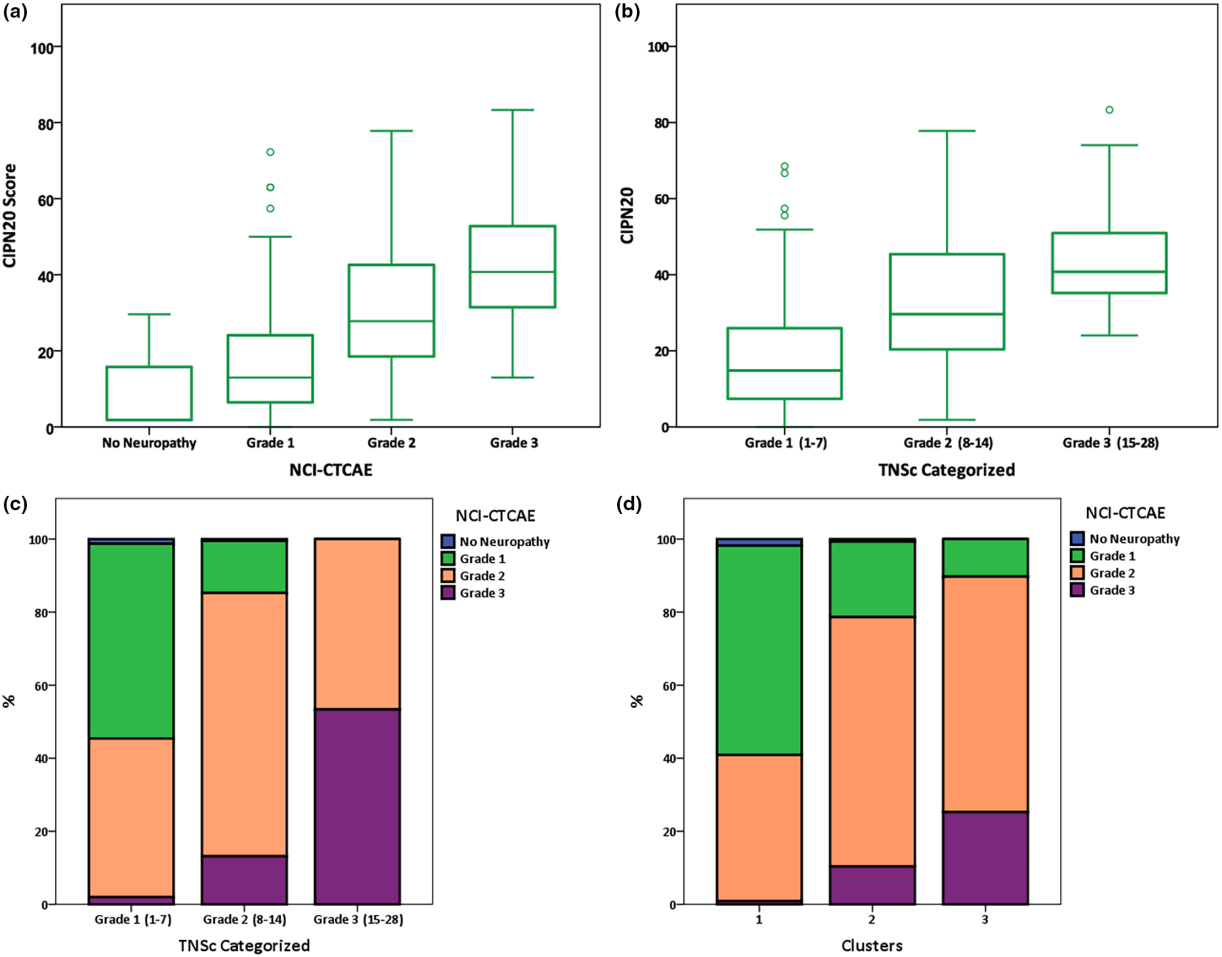
Relationship between neurotoxicity scales and the European Organization for Research and Treatment of Cancer disease‐specific quality of life questionnaire submodule (QLQ‐CIPN20). (a, b) Distribution of neuropathy severity grades measured by National Cancer Institute Common Terminology Criteria for Adverse Events (NCI‐CTCAE; a) and categorized Total Neuropathy Scale–clinical version (TNSc; b) in relation to QLQ‐CIPN20 scores. (c, d) Discrepancies between the categorization of TNSc severity grades and NCI‐CTCAE grades (c) and clusters and NCI‐CTCAE grades (d).

Baseline demographic and clinical characteristics of 372 subjects analyzed (281 from the CI‐PeriNomS study and 91 from the Hospital Universitari de Bellvitge–ICO L'Hospitalet study) are summarized in Table 1. To explore the association between TNSc scores and QLQ‐CIPN20, we categorized TNSc severity scores into four grades based on criteria used in other studies [10, 11]. CIPN severity could be Grade 1 (scores 1–7), Grade 2 (scores 8–14), Grade 3 (scores 15–21), or Grade 4 (scores >21). Both NCI‐CTCAE and TNSc demonstrated a significant difference in the distribution of severity grades concerning QLQ‐CIPN20 scores (*H*[2] = 92.06, *p* < 0.001 and *H*[3] = 85.08, *p* = 0.001, respectively). However, a proportion of subjects across different neurotoxic severity grades exhibited overlapping QLQ‐CIPN20 scores, particularly in the Grade 2 category (Figure 1a,b). In addition, the categorization of TNSc presented significant discrepancies with NCI‐CTCAE grades (*H*[3] = 97.99; *p* < 0.001; Figure 1c). Despite an acceptable correlation when TNSc was considered as a continuous variable (*r* = 0.56, *p* < 0.001), 13% of subjects classified as Grade 3 and 45% of subjects classified as Grade 2 according to NCI‐CTCAE fell into lower grade categories according to the categorized TNSc. Conversely, 16% of subjects graded as 1 and 47% of subjects graded as 2 according to NCI‐CTCAE were classified in higher severity levels according to the TNSc grading.

**TABLE 1:** Demographic and clinical characteristics and prior treatment of participants with their neuropathy grades and QLQ‐CIPN20 scores.

Characteristic	Value
Study population, *N*	372
Age, years, mean ± SD	61.3 ± 10.7
Sex, female, %	48.9
Cancer type, %	
Gastrointestinal tract	45.8
Breast	18.1
Hematologic	15.4
Urogenital tract	6.2
Lung	5.7
Other sites	8.9
Drug class, %	
Platinum	64.2
Antimicrotubule agents^a^	24.7
Proteasome inhibitors	3.8
Thalidomide	2.4
Combination of neurotoxic drugs	4.9
NCI‐CTCAE, %	
No neuropathy^b^	0.8
Grade 1	29
Grade 2	58.4
Grade 3	11.8
TNSc, median (range)	8 (1–20)
Grade 1 (1–7), %	40.9
Grade 2 (8–14), %	51.1
Grade 3 (15–21), %	8.1
SMA subscore, median (range)	3 (0–8)
RVPSt subscore, median (range)	5 (0–13)
QLQ‐CIPN20 score, median (range)	24.1 (0–83.3)

Abbreviations: NCI‐CTCAE, National Cancer Institute Common Terminology Criteria for Adverse Events; QLQ‐CIPN20, European Organization for Research and Treatment of Cancer disease‐specific quality of life questionnaire submodule; RVPSt, reflexes, vibration, pin sensitivity, and muscle strength; SMA, sensory, motor, autonomic; TNSc, Total Neuropathy Scale–clinical version.

^a^ Antimicrotubule agents include mostly taxanes, but also vincristine, epothilones, and brentuximab vedotin.

^b^ Patients without neuropathy according to oncologist evaluation but with chemotherapy‐induced peripheral neurotoxicity according to neurologist assessment.

### Identification of neurotoxic severity patterns

**FIGURE 2:**
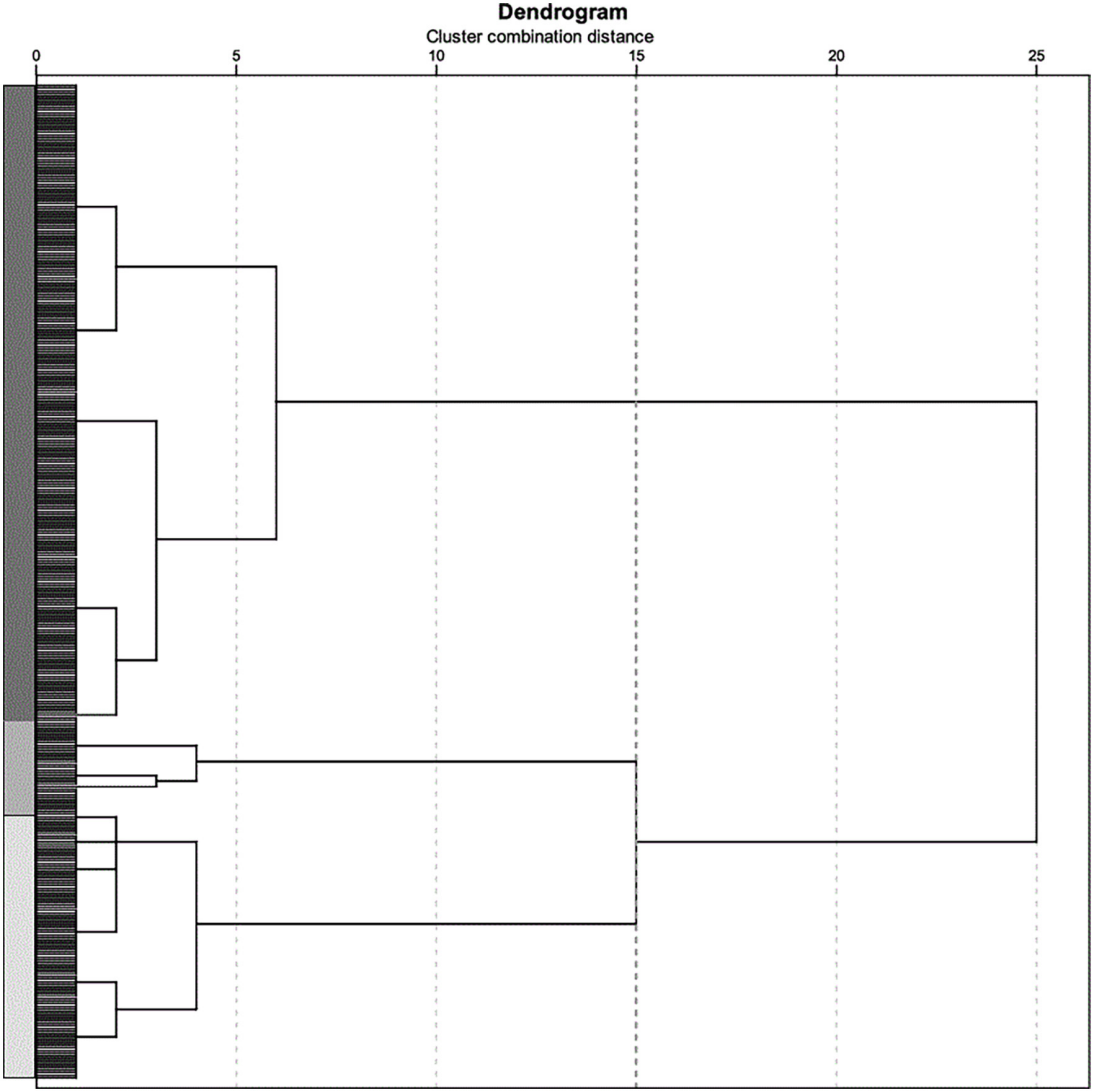
The three cluster solutions revealed in the dendrogram of the hierarchical clustering analysis by visual inspection. The bold dotted line on the cluster combination axis intersects all three potential solutions.

**FIGURE 3:**
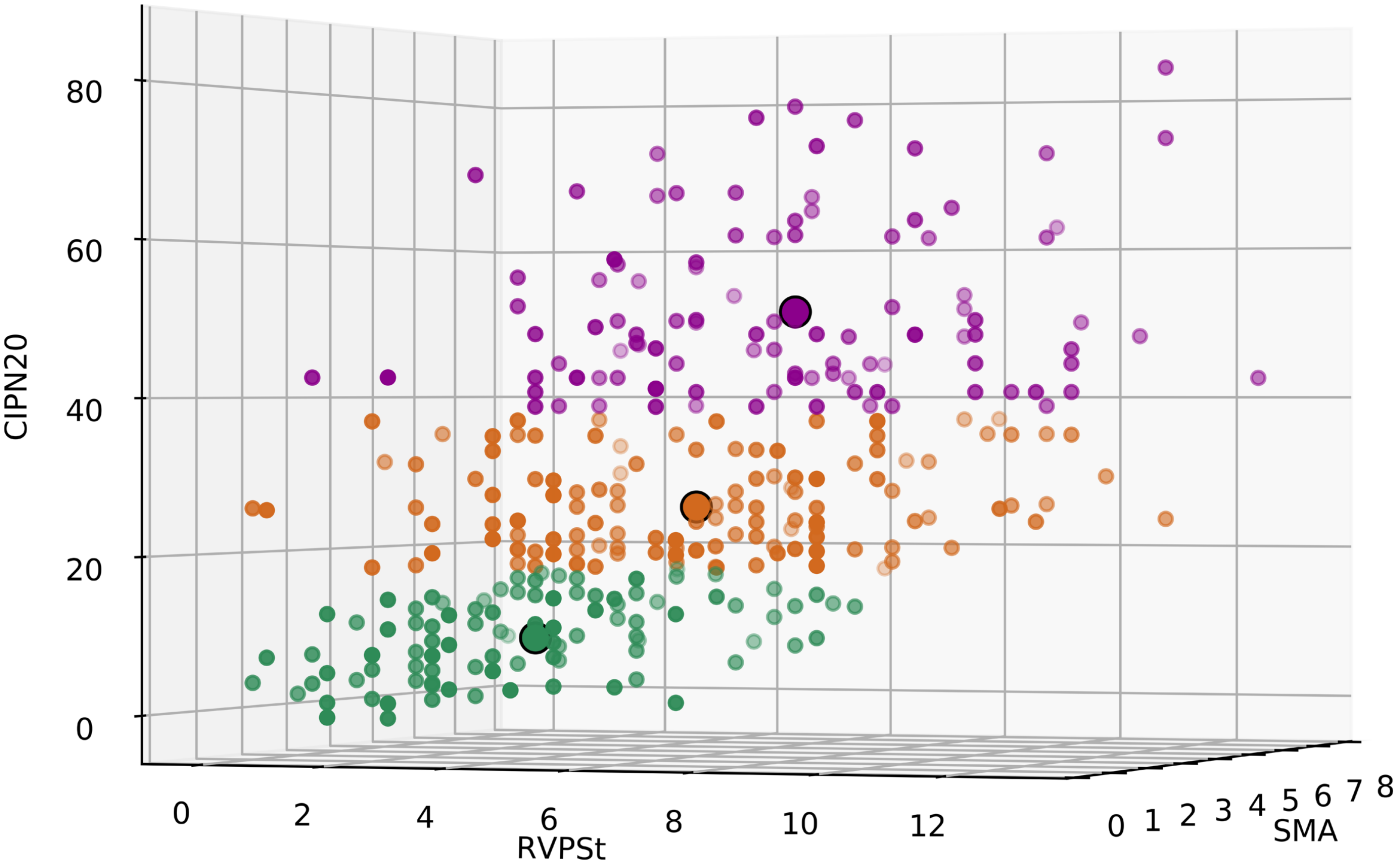
Patients' distribution according to the three‐cluster solution defined by k‐means analysis. Each point corresponds to one patient; bold circles indicate the centroid location. The three‐dimensional space is limited by the neurological symptom axis (sensory, motor and autonomic) and the neurologic sign axis (reflexes, vibration, pin sensitivity, and strength) provided by the Total Neuropathy Scale–clinical version, and the quality of life score axis provided by the European Organization for Research and Treatment of Cancer disease‐specific quality of life questionnaire submodule (QLQ‐CIPN20).

The visual inspection of the agglomeration scree plot and dendrogram of the hierarchical clustering analysis, incorporating TNSc subscores (symptoms and signs) and QLQ‐CIPN20 scores as variables, revealed a three‐cluster solution (Figure 2) according to the cluster combination distance. Subsequently, the k‐means cluster solution defined three clusters in a three‐dimensional space limited by the SMA, RVPSt, and QLQ‐CIPN20 axes. The centroids for each cluster were located at values of 2, 4, and 9.38 for Group 1; 3, 6, and 25.98 for Group 2; and 4, 7, and 51.15 for Group 3 (Figure 3). These clusters represented varying degrees of neurological impairment and quality of life assessment, ranging from less impaired (Group 1, with 110 subjects) to intermediately impaired (Group 2, with 145 subjects) to more impaired (Group 3 with 107 subjects). Vectorial distances between centroids for Group 1 to Groups 2 and 3 were 16.78 and 41.92, and the distance between centroids for Group 2 to Group 3 was 25.21. This cluster solution achieves a good separation between the newly identified groups and the distribution of CIPN20 scores (Figure S1).

The DFA plot of the final k‐means cluster solution revealed cohesive clusters, concentrating subjects around each of the three distinct centroids (Figure S2). DFA produced two discriminant functions explaining 99.1% and 0.9% of the variance, respectively (Wilks lambda = 0.17, *χ* ^2^[6] = 645.47, canonical correlation = 0.91, *p* < 0.001; and Wilks lambda = 0.96, *χ* ^2^[2] = 15.74, canonical correlation = 0.21, *p* < 0.001). These significant results indicate that the functions utilizing the predictive variables SMA, RVPSt, and QLQ‐CIPN20 effectively explain group membership. The analysis further demonstrated that 93.6% of subjects were correctly classified within Group 1, 98.6% within Group 2, and 93.5% within Group 3.

### Cluster comparisons on relationships with other variables

Table 2 provides a comparison of the three clusters in terms of age, sex, drug class received, and cancer type. No statistically significant differences were observed in age, sex, or cancer type among the clusters. Across all three clusters, the mean age was close to 61 years, the male: female ratio was approximately 1:1, and the main cancer types were gastrointestinal and breast. There was a trend for a difference according to class of drug used, but it did not reach statistical significance, with higher platinum use in Cluster 3 and proteasome inhibitors in Cluster 1. Moreover, when the analyses was restricted to the agents received by 95% of subjects (platinum and antimicrotubule agents), the relationship continued to be nonsignificant (*χ* ^2^[2] = 2.44, *p* = 0.3).

**TABLE 2:** Demographic and clinical characteristics and prior treatment of participants as per the *k*‐means three‐cluster solution.

Characteristic	Cluster 1, *n* = 110	Cluster 2, *n* = 145	Cluster 3, *n* = 107	*p*
Age, years, mean ± SD	60.9 ± 11.7	60.9 ± 10.6	62.1 ± 10.4	1
Sex, female, %	45.5	49.7	53.3	0.52
Cancer type, %				
Gastrointestinal tract	43.6	43.4	50.5	0.85
Breast	18.2	19.3	17.8	
Hematologic	22.7	12.4	11.2	
Urogenital tract	5.5	7.6	5.6	
Lung	3.6	6.2	6.5	
Other sites	6.4	11	8.4	
Drug class, %				
Platinum	56.9	63.9	71	0.089
Antimicrotubule agents	29.4	24.3	22.4	
Proteasome inhibitors	8.3	2.8	0	
Thalidomide	0	4.2	1.9	
Combination of neurotoxic drugs	5.5	4.9	5.5	

^a^ Antimicrotubule agents include mostly taxanes, but also vincristine, epothilones, and brentuximab vedotin.

There was a moderate correlation between Clusters 1, 2, and 3 and the adverse event terminology of Grade 1, 2, and 3 for NCI‐CTCAE grades (*r* = 0.46, *p* < 0.001); however, in a cross‐table comparison by grades and cluster groups, a significant number of subjects did not align between these two classifiers (*H*[3] = 76.6, *p* < 0.001; Figure 1d). Specifically, 37.2% of subjects classified as Grade 3 and 20.8% of subjects classified as Grade 2 according to NCI‐CTCAE were categorized in lower grade categories as per the cluster grouping. Conversely, 39.3% of subjects graded as 1% and 32.5% of subjects graded as 2 according to NCI‐CTCAE were assigned higher severity grades based on the cluster classification (see also Figure S3).

## DISCUSSION

Despite being a well‐known adverse event, CIPN persists as a challenge in cancer treatment, impacting patient well‐being and treatment decisions. The differences in the CROs used to measure CIPN severity and the complex interplay between these scales and PROs can contribute to divergent management approaches among neurologists and oncologists. Although current recommendations advocate for the combined use of CROs and PROs for assessing patients undergoing neurotoxic treatments [1, 12], translating this into practical daily practice during ongoing treatment remains challenging.

Our study underscores discrepancies and limitations in existing clinical assessment methods, aligning with findings from other studies [1, 2, 13], and emphasizes the need for a more holistic approach to evaluating CIPN severity, in line with patient preferences [14]. Notably, neither the oncology gold standard NCI‐CTCAE nor the neurology gold standard TNSc are able to adequately capture the complex interplay between patients' experiences and the impact of neuropathy on their lives. This is particularly the case in intermediate toxicity grades (Grade 2), where crucial treatment decisions are made regarding treatment continuation, dose adjustments, or discontinuation, potentially determining long‐lasting quality of life and survival outcomes. Recognizing that even though both the neurological examination and the neuropathy grading can experience improvements over time after treatment completion, there is a substantial proportion of patients who endure lasting neurological impairments and compromised quality of life even after treatment completion [15, 16, 17, 18, 19, 20], underscoring the need for a nuanced assessment approach. This underscores the need to consider patients' experiences in assessing neurotoxicity beyond what current scales capture.

Any changes to the evaluation of CIPN severity must consider the challenge of translating objective neurological findings into clinically meaningful categories. Our group reported on the clinical implications of changes in TNSc scores by identifying the changes in TNSc scores corresponding to the minimal clinically important change as measured by the Functional Assessment of Cancer Treatment/Gynecologic Oncology Group–Neurotoxicity [21]. This can help design clinical trials focused on neuroprotective or proregenerative interventions. However, although this contributes to the understanding of CIPN, translating these findings into CIPN severity categories for use in clinical practice remains a challenge. Our study aimed to bridge this gap by introducing an approach that uses existing tools that are both accessible and practical for patient management in routine clinical practice.

The application of clustering techniques to our dataset revealed three distinct neurotoxic severity patterns based on TNSc variables and QLQ‐CIPN20 scores, providing a novel perspective on patient stratification that captures variations in neurological impairment and quality of life. The DFA further validated the robustness of these clusters, successfully classifying subjects by their respective severity patterns. This approach offers a potential framework for individualizing patient care, enabling tailored interventions based on specific neurotoxic profiles, especially in patients treated with platinum‐based and antimicrotubule agents. To aid implementation, Appendix S2 includes a calculator for rapid patient classification based on the TNSc items from the neurological examination and the QLQ‐CIPN20 answers from patients. The calculator serves as a practical solution for guiding treatment decisions, adhering to the Common Terminology structure for reporting adverse events, while ensuring interventions are aligned with the unique neurotoxic patterns of individual patients. Overall, our study not only contributes to understanding CIPN but also provides a practical tool for real‐world patient management.

The study has several strengths, including its prospective nature, multisite participation, and analysis of a large, diverse sample exposed to commonly used neurotoxic cytostatic drugs. Each of the three different clusters of CIPN severity had a reasonable number of patients, with no significant differences in demographics or clinical characteristics that could impact the CIPN severity or the quality of life. Altogether, these strengths provide robustness and greater generalizability to the results. Study limitations include a predominantly Western European population that may introduce cultural bias, especially regarding patients' experiences collected in QLQ questionnaires. Additionally, and importantly, there is no a priori optimal way to integrate the data complexity from PROs and CROs. Clustering analysis techniques have inherent limitations, such as that a k‐means cluster algorithm assumes spherical cluster shapes, among others, which may not accurately represent the underlying data distribution; however, the internal validity and robustness of our clusters is supported by DFA results. Finally, another potential limitation comes from the greater investment of time needed to apply this tool to assess patients with regard to NCI‐CTCAE, and the short previous training time to use it, depending on the care‐provider specialty formation. To facilitate application of the tool, we supply a calculator (Appendix S2), and we suggest its application for doubtful situations and to assist decision‐making for patients who require dose modifications according to the NCI‐CTCAE scale.

A logical next step for work on this issue would be a prospective clinical trial or study comparing the NCI‐CTCAE neuropathy grading with our cluster‐based approach in terms of patient‐reported and cancer outcomes. As neurotoxic drugs continue to be pivotal in treating various cancers, addressing the challenge posed by CIPN requires collaborative efforts and the establishment of a unified grading system aligned with patient needs. This step is crucial for enhancing the quality of cancer care and addressing the unresolved challenges posed by CIPN.

## AUTHOR CONTRIBUTIONS

**R. Velasco:** Formal analysis; data curation; funding acquisition; investigation; writing – review and editing. **A. A. Argyriou:** Writing – original draft; investigation; supervision; writing – review and editing. **D. R. Cornblath:** Writing – original draft; writing – review and editing; supervision. **P. Bruna:** Methodology; writing – review and editing. **P. Alberti:** Data curation; writing – review and editing; investigation. **E. Rossi:** Data curation; writing – review and editing; investigation. **S. J. Merkies:** Writing – review and editing; investigation. **D. Psimaras:** Writing – review and editing; investigation. **C. Briani:** Writing – review and editing; investigation. **R. I. Lalisang:** Writing – review and editing; investigation. **A. Schenone:** Writing – review and editing; investigation. **G. Cavaletti:** Writing – original draft; writing – review and editing; supervision; investigation. **J. Bruna:** Conceptualization; methodology; formal analysis; writing – original draft; writing – review and editing; funding acquisition; supervision; investigation.

## FUNDING INFORMATION

R.V. has received support from the Instituto de Salud Carlos III, through the project PI20/00283 (cofunded by the European Regional Development FUND‐ERDF), and the Spanish Foundation Research Group in Breast Cancer (GEICAM). J.B. was supported by a PI21/00181 grant from the Instituto de Salud Carlos III of Spain, cofunded by the European Union (ERDF/ESF, “Investing in Your Future”). We thank the CERCA Programme/Generalitat de Catalunya for institutional support.

## CONFLICT OF INTEREST STATEMENT

The authors declare no conflict of interest.

## Supporting information


Figure S1.

Figure S2.

Figure S3.

Appendix S1.

Appendix S2.

## Data Availability

The data that support the findings of this study are available from the corresponding author upon reasonable request.
