# Supplementary figures and images for: Repurposing chemotherapy‐induced peripheral neuropathy grading
Source: Eur J Neurol. 2024 Sep 16;31(12):e16457. doi: 10.1111/ene.16457 (PMC11554987; doi:10.1111/ene.16457)

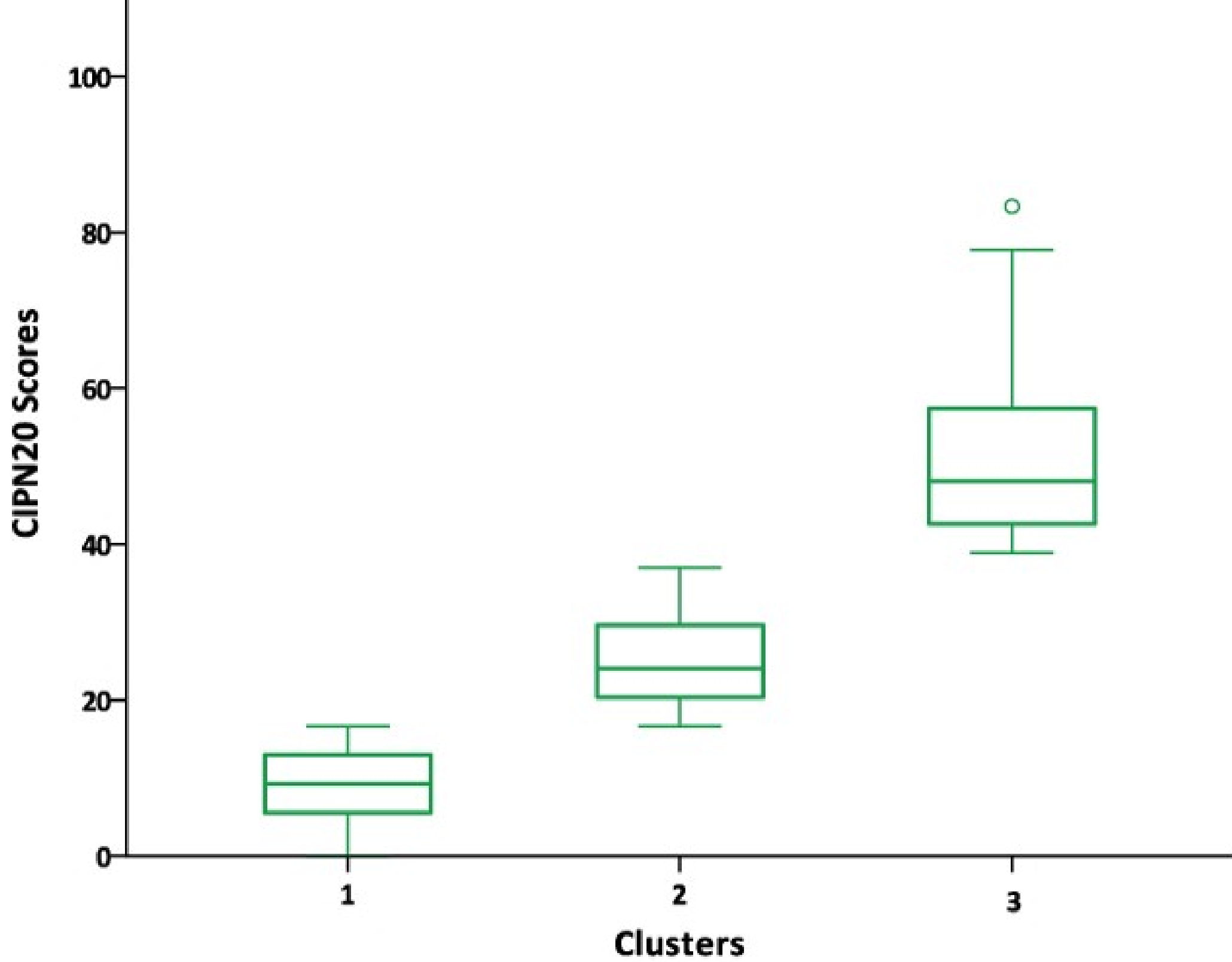

Supplement: Supplementary file 1 — Figure S1. [file ENE-31-e16457-s004.pdf]

# Discriminant Canonical Functions

Function 2

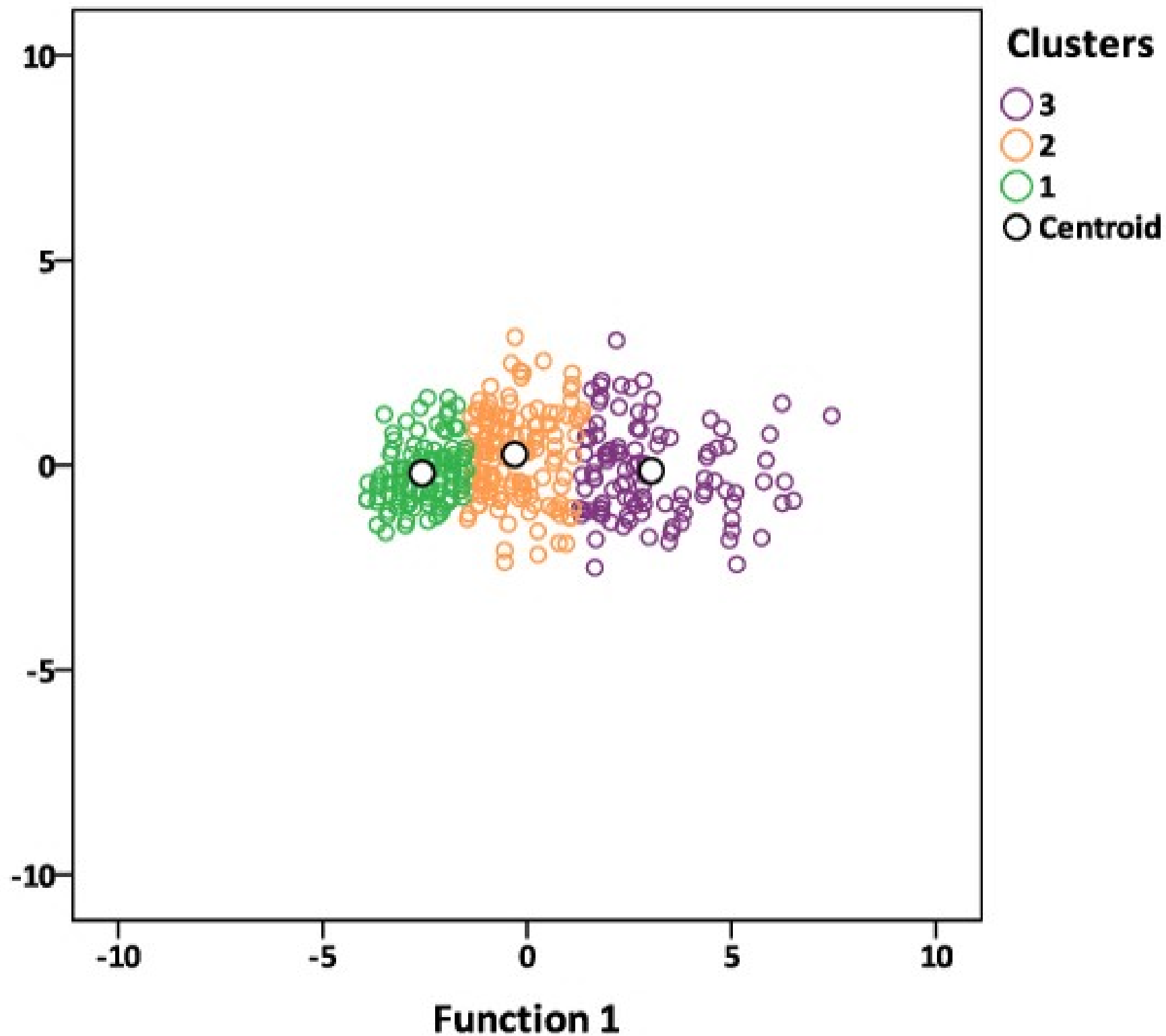

Supplement: Supplementary file 2 — Figure S2. [file ENE-31-e16457-s005.pdf]
