# Supplementary material for: Repurposing chemotherapy‐induced peripheral neuropathy grading
Source: Eur J Neurol. 2024 Sep 16;31(12):e16457. doi: 10.1111/ene.16457 (PMC11554987; doi:10.1111/ene.16457)
Supplement: Supplementary file 3 — Figure S3. [file ENE-31-e16457-s001.pdf]

NCI-CTCAE Grades

TNSc-CIPN20 Cluster Groups

	0-1	2	3	Nº of pa[illegible]
1	65<br>60.7%	44<br>20.8%	1<br>2.3%	11[illegible]
2	31<br>29%	99<br>46.7%	15<br>34.9%	14[illegible]
3	11<br>10.3%	69<br>32.5%	27<br>62.8%	10[illegible]
	107	212	43	36[illegible]

- (green) Agreements between the different grades grading systems
- (pink) CTCAE classified patients who would present lower grades according to the cluster groupin[illegible]
- (purple) CTCAE classified patients who would present higher grades according to the cluster groupin[illegible]

*Ten patients missed t[illegible]
