# Supplementary material for: Repurposing chemotherapy‐induced peripheral neuropathy grading
Source: Eur J Neurol. 2024 Sep 16;31(12):e16457. doi: 10.1111/ene.16457 (PMC11554987; doi:10.1111/ene.16457)
Supplement: Supplementary file 4 — Appendix S1. [file ENE-31-e16457-s002.docx]

STROBE Statement—checklist of items that should be included in reports of observational studies

|  | Item No. | Recommendation | Page  No. | Relevant text from manuscript |
| --- | --- | --- | --- | --- |
| **Title and abstract** | 1 | (*a*) Indicate the study’s design with a commonly used term in the title or the abstract |  |  |
|  |  | (*b*) Provide in the abstract an informative and balanced summary of what was done and what was found | Page 5 | Data were pooled from two prospective studies |
| Introduction | | | |  |
| Background/rationale | 2 | Explain the scientific background and rationale for the investigation being reported | Page 7 | See Introduction |
| Objectives | 3 | State specific objectives, including any prespecified hypotheses | Page 7 | “identify distinct patient clusters based on TNSc variables and quality of life test scores”  “aims to facilitate the development of a pragmatic severity grade categorization that can effectively guide clinical decisions “ |
| Methods | | | |  |
| Study design | 4 | Present key elements of study design early in the paper | Page 7  Page 8 | Cross-sectional?  Study design |
| Setting | 5 | Describe the setting, locations, and relevant dates, including periods of recruitment, exposure, follow-up, and data collection | Page 7 | Introduction: multicentre European study |
| Participants | 6 | (*a*) *Cohort study*—Give the eligibility criteria, and the sources and methods of selection of participants. Describe methods of follow-up  *Case-control study*—Give the eligibility criteria, and the sources and methods of case ascertainment and control selection. Give the rationale for the choice of cases and controls  *Cross-sectional study*—Give the eligibility criteria, and the sources and methods of selection of participants | Page 8 | Materials and Methods  Patient Sample  Inclusion and exclusion criteria |
|  |  | (*b*) *Cohort study*—For matched studies, give matching criteria and number of exposed and unexposed  *Case-control study*—For matched studies, give matching criteria and the number of controls per case |  | - |
| Variables | 7 | Clearly define all outcomes, exposures, predictors, potential confounders, and effect modifiers. Give diagnostic criteria, if applicable | Page 8 | See details on:  Assessment methods |
| Data sources/ measurement | 8* | For each variable of interest, give sources of data and details of methods of assessment (measurement). Describe comparability of assessment methods if there is more than one group |  |  |
| Bias | 9 | Describe any efforts to address potential sources of bias |  |  |
| Study size | 10 | Explain how the study size was arrived at | Page 8 | 372 patients were included in our study: 281 from the CI-PeriNomS study and 91 |

Continued on next page

| Quantitative variables | 11 | Explain how quantitative variables were handled in the analyses. If applicable, describe which groupings were chosen and why | Page 10 |  |
| --- | --- | --- | --- | --- |
| Statistical methods | 12 | (*a*) Describe all statistical methods, including those used to control for confounding | Page 9-10 | See details on :  Cluster and Statistical Analysis |
|  |  | (*b*) Describe any methods used to examine subgroups and interactions | Page 10 | To explore the association between TNSc scores and QLQ-CIPN20, we categorized TNSc severity scores into four grades based on criteria used in other studies.10,11 CIPN severity could be Grade 1 (scores 1–7), Grade 2 (scores 8–14), Grade 3 (scores 15–21), and Grade 4 (scores >21). |
|  |  | (*c*) Explain how missing data were addressed |  |  |
|  |  | (*d*) *Cohort study*—If applicable, explain how loss to follow-up was addressed  *Case-control study*—If applicable, explain how matching of cases and controls was addressed  *Cross-sectional study*—If applicable, describe analytical methods taking account of sampling strategy | Page 10-11 | The inspection of the agglomeration scree plot and dendrogram of the hierarchical clustering analysis, incorporating TNSc subscores (symptoms and signs) and QLQ-CIPN20 scores as variables, revealed a three-cluster solution (Figure 2). |
|  |  | (*e*) Describe any sensitivity analyses |  |  |
| Results | | | | |
| Participants | 13* | (a) Report numbers of individuals at each stage of study—eg numbers potentially eligible, examined for eligibility, confirmed eligible, included in the study, completing follow-up, and analysed | Page 8 | Patient sample  372 patients were included in our study: 281 from the CI-PeriNomS study and 91 from the Hospital Universitari de Bellvitge-ICO L’Hospitalet study |
|  |  | (b) Give reasons for non-participation at each stage | Page 8 | Our study included those patients who had stable CIPN and had undergone assessments related to quality of life, neurological symptoms, and NCI-CTCAE evaluations |
|  |  | (c) Consider use of a flow diagram |  |  |
| Descriptive data | 14* | (a) Give characteristics of study participants (eg demographic, clinical, social) and information on exposures and potential confounders | Page 10 | Baseline demographic and clinical characteristics of 372 patients analyzed in table 1 |
|  |  | (b) Indicate number of participants with missing data for each variable of interest |  |  |
|  |  | (c) *Cohort study*—Summarise follow-up time (eg, average and total amount) |  |  |
| Outcome data | 15* | *Cohort study*—Report numbers of outcome events or summary measures over time |  |  |
|  |  | *Case-control study—*Report numbers in each exposure category, or summary measures of exposure |  |  |
|  |  | *Cross-sectional study—*Report numbers of outcome events or summary measures | Pages 10-12 | Relationship between Neurotoxicity Scales and CIPN20 QLQ  Identification of Neurotoxicity Severity Patterns  Cluster Comparisons on Relationships with Other Variables |
| Main results | 16 | (*a*) Give unadjusted estimates and, if applicable, confounder-adjusted estimates and their precision (eg, 95% confidence interval). Make clear which confounders were adjusted for and why they were included |  |  |
|  |  | (*b*) Report category boundaries when continuous variables were categorized | Page 11 | Table 2 provides a comparison of the three clusters in terms of age, sex, drug class received, and cancer types |
|  |  | (*c*) If relevant, consider translating estimates of relative risk into absolute risk for a meaningful time period |  |  |

Continued on next page

| Other analyses | 17 | Report other analyses done—eg analyses of subgroups and interactions, and sensitivity analyses |  |  |
| --- | --- | --- | --- | --- |
| Discussion | | | | |
| Key results | 18 | Summarise key results with reference to study objectives | Page 12 | “Our study underscores discrepancies and limitations in existing clinical assessment methods”  “Our study aimed to bridge this gap, by introducing an approach that uses existing tools that are both accessible and practical for patient management in routine clinical practice”  “The application of clustering techniques to our dataset revealed three distinct neurotoxic severity patterns based on TNSc variables and CIPN20 scores, providing a novel perspective on patient stratification that captures variations in neurological impairment and quality of life”  “our study contributes not just to understanding CIPN but also provides a practical tool for real-world patient management” |
| Limitations | 19 | Discuss limitations of the study, taking into account sources of potential bias or imprecision. Discuss both direction and magnitude of any potential bias | Page 13 | Study limitations include a predominantly West-European population that may introduce cultural bias, especially regarding patients' experiences collected in QLQs. Additionally, clustering analysis techniques have complexity and inherent limitations, such as k-means cluster algorithm assumes spherical cluster shapes, among others, which may not accurately represent the underlying data distribution; but the internal validity and robustness of our clusters is supported by DFA results. |
| Interpretation | 20 | Give a cautious overall interpretation of results considering objectives, limitations, multiplicity of analyses, results from similar studies, and other relevant evidence | Page 12-13 | Discussion |
| Generalisability | 21 | Discuss the generalisability (external validity) of the study results | Page 14 | A logical next step would be a prospective clinical trial comparing the NCI-CTCAE neuropathy grading with our cluster-based approach in terms of patient-reported and cancer outcomes. As neurotoxic drugs continue to be pivotal in treating various cancers, addressing the challenge posed by CIPN requires collaborative efforts and the establishment of a unified grading system aligned with patient needs. |
| Other information | |  | | |
| Funding | 22 | Give the source of funding and the role of the funders for the present study and, if applicable, for the original study on which the present article is based | Page 14 | Funding |

*Give information separately for cases and controls in case-control studies and, if applicable, for exposed and unexposed groups in cohort and cross-sectional studies.

**Note:** An Explanation and Elaboration article discusses each checklist item and gives methodological background and published examples of transparent reporting. The STROBE checklist is best used in conjunction with this article (freely available on the Web sites of PLoS Medicine at http://www.plosmedicine.org/, Annals of Internal Medicine at http://www.annals.org/, and Epidemiology at http://www.epidem.com/). Information on the STROBE Initiative is available at www.strobe-statement.org.
